# Ionic Mechanisms of Endogenous Bursting in CA3 Hippocampal Pyramidal Neurons: A Model Study

**DOI:** 10.1371/journal.pone.0002056

**Published:** 2008-04-30

**Authors:** Jun Xu, Colleen E. Clancy

**Affiliations:** Department of Physiology and Biophysics, Institute for Computational Biomedicine, Weill Medical College of Cornell University, New York, New York, United States of America; University of Southern California, United States of America

## Abstract

A critical property of some neurons is burst firing, which in the hippocampus plays a primary role in reliable transmission of electrical signals. However, bursting may also contribute to synchronization of electrical activity in networks of neurons, a hallmark of epilepsy. Understanding the ionic mechanisms of bursting in a single neuron, and how mutations associated with epilepsy modify these mechanisms, is an important building block for understanding the emergent network behaviors. We present a single-compartment model of a CA3 hippocampal pyramidal neuron based on recent experimental data. We then use the model to determine the roles of primary depolarizing currents in burst generation. The single compartment model incorporates accurate representations of sodium (Na^+^) channels (Na_V_1.1) and T-type calcium (Ca^2+^) channel subtypes (Ca_V_3.1, Ca_V_3.2, and Ca_V_3.3). Our simulations predict the importance of Na^+^ and T-type Ca^2+^ channels in hippocampal pyramidal cell bursting and reveal the distinct contribution of each subtype to burst morphology. We also performed fast-slow analysis in a reduced comparable model, which shows that our model burst is generated as a result of the interaction of two slow variables, the T-type Ca^2+^ channel activation gate and the Ca^2+^-dependent potassium (K^+^) channel activation gate. The model reproduces a range of experimentally observed phenomena including afterdepolarizing potentials, spike widening at the end of the burst, and rebound. Finally, we use the model to simulate the effects of two epilepsy-linked mutations: R1648H in Na_V_1.1 and C456S in Ca_V_3.2, both of which result in increased cellular excitability.

## Introduction

A hallmark of CA3 hippocampal neurons is intrinsic burst firing. In primates 95% of CA3 neurons burst [1], while in rodents distinct populations of bursting and non-bursting CA3 neurons have been identified [2]. Synapses in the central nervous system are notoriously fickle in transmitting information and bursting may improve the reliability of information transmission by facilitating transmitter release [3]. However, the delicate balance of currents that produces an endogenous burst in a single neuron may also contribute to the susceptibility of networks of bursting cells to debilitating recurrent excitation.

### The endogenous cellular burst and the network burst

In a network of neurons synchronous bursting causes seizures [4], a hallmark of epilepsy. Unlike an endogenous burst in a single neuron, synchronous bursting in a population depends on synaptic interactions between neurons. The cellular epileptic waveform resulting from synaptic interactions is the paroxysmal depolarizing shift (PDS), a waveform that is distinct from the endogenous single cell burst mediated by the active membrane properties in an individual cell [5]. Network bursts and endogenous bursts are nonetheless associated; the propensity of CA3 neurons to fire bursts of four to five action potentials may increase 10-fold the probability of recruiting synaptically connected neurons to burst [5], and the synaptic integration required for network transmission is mediated by active conductances in the membrane. Therefore, understanding the ionic mechanisms of CA3 bursting is important to determine the mechanisms of synchronized behavior in neuronal networks.

Here we present the first step in that direction by developing a single-compartment model to represent the CA3 soma that incorporates recent data on primary depolarizing currents in CA3 hippocampal neurons. We then use the model to suggest ionic mechanisms of endogenous bursts and predict the effect of the naturally occurring epilepsy associated Na_V_1.1 R1648H and Ca_V_3.2 C456S mutations on cellular electrical activity [6], [7], [8], [9], [10].

### Ionic mechanisms of endogenous bursts

Sodium (Na^+^) and calcium (Ca^2+^) currents contribute to bursting in CA3 neurons [11], [12], [13], [14], although specific contributions from subtypes of Na^+^ and Ca^2+^ channels are not known. A primary component of the endogenous burst in CA3 neurons is the afterdepolarizing potential (ADP), which is observed as a persistent depolarization, or incomplete repolarization, following fast spike depolarizations during the burst. Multiple cell-type specific ionic mechanisms underlying the afterdepolarizing potential (ADP) have been suggested. Studies have shown that Ca^2+^ current [11], [12], [13], [15], persistent Na^+^ current [16], both persistent Na^+^ and T-type Ca^2+^ currents [17], or the spatial-temporal interactions between soma and dendrite (the “ping-pong” effect) [18] can contribute to the generation of afterdepolarizing potentials (ADPs) and trigger burst firing.

In this study, we incorporate our previously published model of an individual Na^+^ channel and newly developed models of Ca^2+^ channel subtypes in CA3 neurons and use simulations to determine their contribution to the burst waveform. We previously developed detailed Markov models of cardiac and neuronal Na^+^ channels, to overcome limitations of Hodgkin-Huxley models such as the representation of activation and inactivation gating as independent entities, and to simulate mutations that affect discrete kinetic transitions [19], [20]. Here, we use this published model framework for the somatic neuronal Na^+^ channel Na_V_1.1 present in CA3 [20].

By using the Markov model, we better approximate experimentally measured channel properties. An important result of this is a reduction in the window current that was prominent and critical (and artificial) for burst generation in previous models [21], [22], [23]. The window current results from the large overlap of steady-state inactivation and activation curves and may be partially an artifact of the Hodgkin-Huxley Na^+^ channel representation used in previous models [21], [22]. Incorporation of Markov models also allows for functional effects of epilepsy-linked Na^+^ channel mutations that affect discrete transitions to be explicitly represented [6], [7], [20].

Experiments suggest that Ca^2+^ channels are abundant in CA3 neurons and contribute to bursting [24], [25], [26], [27]. We focused on three types of low-voltage-activated T-type Ca^2+^ channels that were recently identified in CA3 neurons [28], since they have been implicated in afterdepolarizing potential (ADP) generation in Purkinje and thalamic relay neurons [29]. The availability of these new data allows us to incorporate a separate channel model for each individual subtype, in contrast to previous computational studies where low-voltage-activated Ca^2+^ channels were lumped into a single generalized channel model [21], [30]. In doing so, we can make experimentally testable predictions that are specific to the subtypes in CA3 neurons and define the role for each subtype in bursting.

In summary, we present a single-compartment computational model of a CA3 hippocampal neuron [22], with a focus on ion channel kinetics of primary depolarizing currents. By incorporating experimentally based models of multiple subtypes of channels, characteristic action potential behaviors are reproduced by the model including, spike broadening that occurs at the end of a burst and rebound burst firing in response to inhibitory postsynaptic potentials. Fast-slow analysis reveals that bursting in our model is generated as a result of the interaction of two slow variables, a slow autocatalytic variable (T-type Ca^2+^ channel activation gate) and a slow negative feedback variable (Ca^2+^-dependent potassium (K^+^) channel activation gate). We also simulate the effect of the epilepsy-linked mutations on single cell dynamics. By robustly representing the wild-type excitable behavior of a single neuron and the behavior resulting from ion channel mutations associated with epilepsy, this model represents a building block for construction of hippocampal network models towards an understanding of how naturally occurring perturbations lead to emergent epileptic behaviors.

## Methods

### CA3 cell model

The model includes the delayed-rectifier potassium (K^+^) current I_KDR_, the transient K^+^ current I_A_, the M current I_M_, the fast sodium (Na^+^) current I_Na_ (Na_V_1.1), two calcium (Ca^2+^)-dependent K^+^ currents I_AHP_ and I_KC,_ three Ca^2+^ currents: low-threshold I_CaT_ (Ca_V_3.1, Ca_V_3.2, and Ca_V_3.3), I_CaL_, and I_CaN_, a leak current I_Leak._ I_st_ is the applied current. In addition, processes that regulate ionic concentration changes are included [22]. The membrane equation is
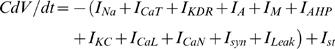
(1)


The maximal conductances are: g_Na1.1_ = 0.07 S/cm^2^, g_CaT_ = 0.45×10^−3^ S/cm^2^, g_KDR_ = 0.08 S/cm^2^, g_A_ = 0.0001 S/cm^2^, g_M_ = 0.00002 S/cm^2^, g_AHP_ = 1.8×10^−6^ S/cm^2^, g_KC_ = 0.00005 S/cm^2^, g_CaL_ = 0.0025 S/cm^2^, g_CaN_ = 0.0025 S/cm^2^, and g_Leak_ = 1/60000 S/cm^2^.

### Channel kinetics

We used a previously published Markov scheme for Na_V_1.1 [20] (see Text S1 and Figure S1 in the online supplement for details). For T-type Ca^2+^ channels, we re-fit parameters to previously published kinetic models of three subtypes of T-type Ca^2+^ channels, based on a recent experimental study, since the parameters from the previous study were not available [29]. For the rate functions and simulated kinetics for T-type Ca^2+^ channel subtypes, please see online supplemental material for details (Text S1 and Figure S2). For Ca^2+^ channel kinetics (N- and L- types), we used available models [30]. For the slow Ca^2+^-activated I_AHP_ current, we adopted a previously published model [31]. The fast Ca^2+^-activated K^+^ current I_KC_, the delayed rectifier K^+^ current I_KDR_, the A-type K^+^ current I_A_, and the muscarinic K^+^ current I_M_ were obtained from a previously published study [22].

### Ca^2+^ dynamics

The intracellular Ca^2+^ concentration was calculated by dCa^2+^/dt = −2I_Ca_/(rF)−Ca^2+^/|ca, where 2/(rF) = 0.014 (radius for soma is 15 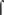
m) is the factor to convert surface Ca^2+^ flux into concentration and op |ca = 50 ms is the time constant for Ca^2+^ removal due to buffering and pumping.

### Method for fast-slow analysis

Fast-slow analysis was used to investigate the bursting mechanism in a reduced single-compartment (soma) CA3 model [32]. Based on this method, bursting mechanisms can be classified into several schemes that rely on the interplay between an autocatalytic depolarization process and slower negative feedback [32]. Our model falls into the “parabolic bursting” category defined by the presence of at least two slow variables: a slow autocatalytic process (T-type Ca^2+^ channels) and a slow negative feedback (Ca^2+^ activated K^+^ current). We used a reduced model that allows for separation of the variables of interest into two subsystems: fast (completed during a spike) and slow (completed over the time scale of burst duration). In our model, the slow autocatalytic variable is n (T-type Ca^2+^ channel activation gate) and the slow negative feedback variable is o (Ca^2+^-dependent K^+^ channel activation gate); all other variables are considered fast. The simplified membrane equation is:
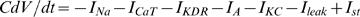
(2)Where the ionic currents are defined as before except the Na^+^ channel is reformulated as a Hodgkin-Huxley model fit to the experimental inactivation and activation curves [6]. We assume steady-state activation m = m_∞_ =  1/(1+exp(−(v+26.4)/7.1)), steady-state inactivation h = h_∞_ = 1/(1+exp((v+67.5)/6.2)), and the time constant for inactivation τ_h_ = 0.197+10.701/(1+exp((v+78.87)×0.0538)). The maximal conductances are: g_Na_ = 2.0 S/cm^2^, g_CaT_ varies between 0.1×10^−3^ and 2×10^−3^ S/cm^2^, g_KDR_ = 0.08 S/cm^2^, g_A_ = 0.0001 S/cm^2^, g_KC_ = 0.0004 S/cm^2^, and g_Leak_ = 1/60000 S/cm^2^. The steady current injection is 0.02 nA. The nonlinear dynamics package XPPAUT was used to compute the fast-slow diagrams [33].

### Computer programming and numerical simulations

Simulations were performed using the explicit Euler method. We used a time step of 10 µs, and further reducing the time step did not generate differences, indicating that temporal accuracy was obtained. We used R_m_ = 60000 Ω cm^2^ (membrane resistivity) and C_m_ = 1 µF/cm^2^. Other parameters were: membrane resting potential V_rest_ = −65 mV, extracellular Ca^2+^ concentration [Ca^2+^]_o_ = 2 mM, intracellular Ca^2+^ concentration [Ca^2+^]_I_ = 50 nM, V_k_ = −91 mV, and V_Na_ = 50 mV. Simulation programs were written in C/C++ programming language and run on a Power Mac G5 dual 2GHz computer. Source code used for simulations in this paper is freely available and can be obtained by emailing clc7003med.cornell.edu.

## Results

We began development of a single-compartment CA3 model neuron by incorporating a model of the voltage gated sodium (Na^+^) channel Na_V_1.1 that we published previously [20] and formulating models of T-type calcium (Ca^2+^) (Ca_V_3.1, Ca_V_3.2 and Ca_V_3.3) channels. Detailed descriptions of the channel models including kinetics and model parameters are contained in the online supplement (see Text S1, Table S1, Table S2, Figure S1 and Figure S2).

### Burst firing in CA3 neurons

**Figure 1:**
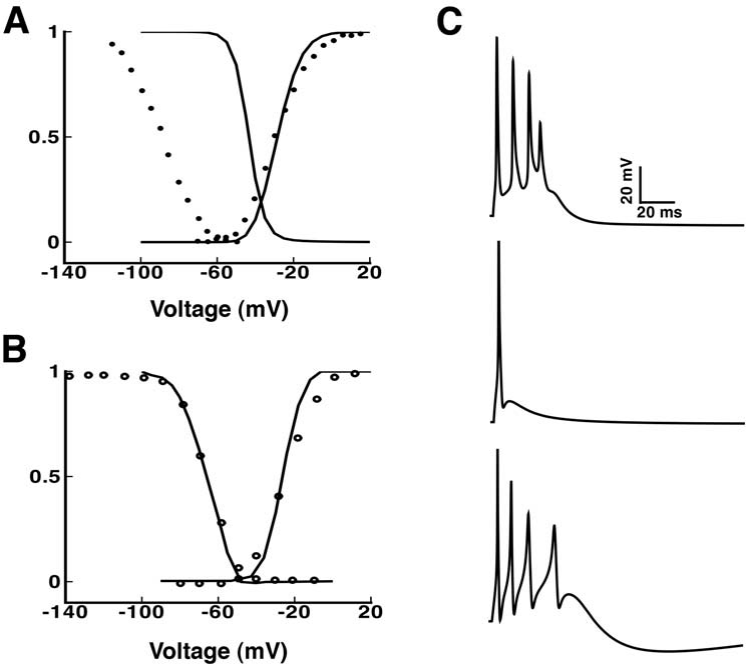
Accurate channel kinetics are important to determine bursting mechanisms. (A) Na^+^ channel activation and steady state inactivation curves generated by the Migliore CA3 model are shown (lines) with superimposed experimental data from Sah et al. [34] on which the model is based (symbols). (B) Activation and steady state inactivation curves generated by the Markov Na_V_1.1 model are shown (lines) with superimposed experimental data from Lossin et al. (6) on which the model is based (symbols). (C) The single compartment model is stimulated with an injection current of 0.2nA for 5 ms. Top panel: Window current resulting from the large artificial rightward shift in the Na^+^ channel inactivation curve results in a burst generated by the Migliore model. Middle panel: Substitution of the Markov Na_V_1.1 model into the Migliore model abolishes the window current, and consequently, the burst. Lower panel: Our CA3 model does not rely on Na^+^ window current for afterdepolarizing potential (ADP) generation. The afterdepolarizing potential (ADP) results from T-type Ca^2+^ currents.

Figure 1 illustrates the importance of incorporating accurate representations of channel kinetics for investigation of bursting mechanisms. The Na^+^ channel model used in the Migliore CA3 model (Figure 1A, solid lines) was based on (and, as acknowledged by the authors, subsequently modified from) experimental data from Sah et al. [34] (Figure 1A, symbols). Superimposition of the activation and inactivation curves generated from the Migliore model onto the experimental data in Figure 1A, shows that the simulated inactivation curve is shifted by >+40 mV compared to the experiment. The effect of this shift is an enormous overlap between activation and inactivation curves, leading to a prominent window current (reviewed in [35]). Our Markov Na_v_1.1 model (solid lines in B) lacks prominent window current and fits the experimental data (symbols in B). Bursting following a 5 ms somatic current injection in the Migliore model is driven by this Na^+^ window current (panel C, top). This is evidenced by the fact that substituting our Markov Na_v_1.1 model for the Migliore Na^+^ channel model abolishes the burst (C, middle panel). The Na^+^ window current supported somatic bursting mechanism is not consistent with the available experimental data for CA3 neurons [12], [13], [25], [26], which suggest that Ca^2+^ current is more critical. Indeed, incorporation of models of the three distinct T-type Ca^2+^ channel subtypes present in CA3 neurons restores bursting even with the windowless Na^+^ current Markov model (C, lower panel). This model simulated the experimentally measured features of a CA3 neuron including overall morphological resemblance, the afterdepolarizing potential (ADP), and the prominent afterhyperpolarization (AHP). The model neuron also reproduced the wide slow spike at the end of the burst.

**Figure 2:**
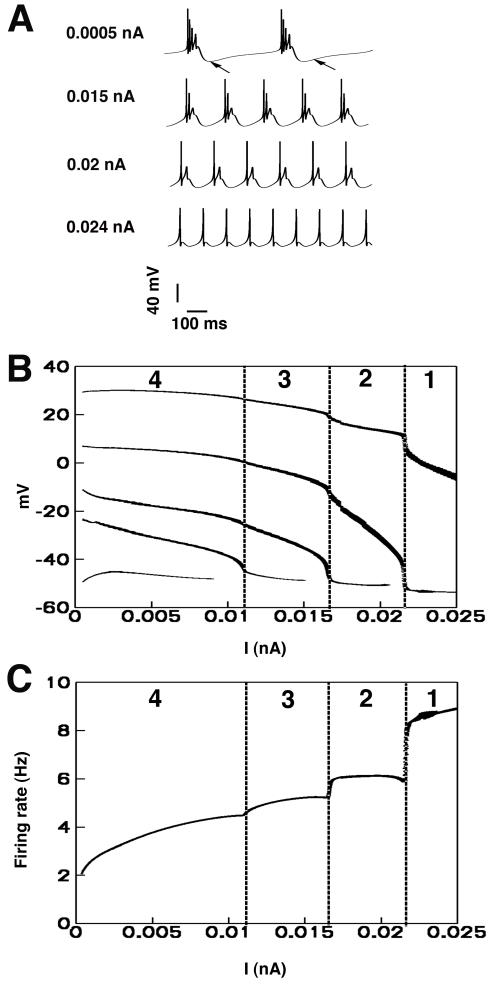
The CA3 neuron exhibits distinct firing modes in response to continuous current injection. (A) Simulated neuronal firing in the CA3 neuron. For smaller amplitude stimulus current, the neuron fires bursts at low frequency (0.0005 nA, 0.015 nA, and 0.02 nA). The burst morphology consistently occurs as a burst of two or more action potentials. With increasing levels of injected stimulus current, the firing becomes regular spiking (0.024 nA). Note that the prominent afterhyperpolarizations observed in experimental recordings are reproduced in the model (Arrows in A). (B) The maximum values of V_m_ for each spike within a burst (recorded at psuedo-steady-state between 9000 ms and 10000 ms) are shown at each value of injection current I (nA). Vertical dashed lines divide the graph into the four zones. The number in each zone indicates the number of spikes in each burst. (C) The intra-burst frequencies are shown at each value of injection current I (nA). Vertical dashed lines divide the graph into the four zones corresponding to those in B. The number in each zone indicates the number of spikes in each burst.

We next examined the effect of changing stimulus duration and amplitude on the response of the model neuron. It has been shown experimentally that steady injection evokes repetitive bursts and higher amplitude stimuli cause increased burst frequency [12], [13]. The model also generated these firing patterns as shown in Figure 2A (recorded at psuedo-steady-state between 9000 ms and 10000 ms). As the stimulus amplitude was further increased in the model, bursts of action potentials disappeared and single spike action potentials were elicited as shown in Figure 2A (bottom panel). This trend is consistent with experimental recordings where increasing amplitude of constant injections gave rise to single spiking modes from bursting modes [12], [13]. In Figure 2B, the peak values of V_m_ for each spike within a burst (recorded at psuedo-steady-state between 9000 ms and 10000 ms) are shown at each value of injection current I (nA). Vertical dashed lines divide the graph into four zones. The number in each zone indicates the number of spikes in each burst. Additionally, the model recapitulated the experimental findings that increasing the stimulus current amplitude leads to an increased intra-burst firing frequency [13] (Figure 2C).

### Rebound excitation

**Figure 3:**
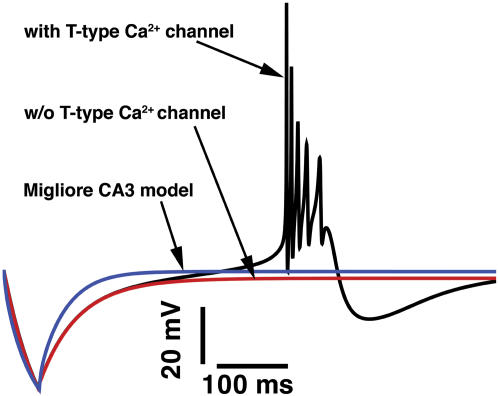
Rebound bursting in the CA3 model neuron. Rebound bursting responses of the CA3 model neuron to a hyperpolarizing current injection (0.03 nA for 50 ms) to mimic inhibitory GABA synaptic inputs. Note the initial hyperpolarization of the membrane and the subsequent rebound before action potential firing (black line). Without T-type Ca^2+^ channels, the model fails to rebound (red line). The Migliore CA3 model is also unable to generate rebound excitation in response to a hyperpolarizing current injection (0.3 nA for 50 ms, blue line).

Next we examined the effects of inhibitory inputs (GABA) on burst firing as illustrated in Figure 3. Our model (Figure 3, black line) simulates the experimentally observed rebound burst [14]. The time course of somatic rebound burst firing in response to a transient hyperpolarizing current injection is shown. The hyperpolarization (<−65mV) recovers T-type Ca^2+^ channels from inactivation. The channels then open and cause a rebound excitation, which brings the membrane to threshold for Na^+^ channels to activate. In the absence of T-type Ca^2+^ channels burst firing is not observed (Figure 3, red line). The Migliore CA3 model is also unsuccessful in generating a rebound burst (Figure 3, blue line), a failure that has been attributed to insufficiently accurate representation of Na^+^ and/or Ca^2+^ channel kinetics [4].

### Na^+^ and T-type Ca^2+^ channels contribute uniquely to the burst

We next utilized the model to ask, “What are the ionic mechanisms that initiate and sustain an action potential burst of longer duration than the trigger?” Bursting in CA3 neurons is known to depend on Na^+^ and Ca^2+^ channels [4], [11], [12], [13]. Tetrodotoxin (TTX) application or inactivation of Na^+^ channels by depolarization abolishes fast spikes. Ca^2+^ channel blockers prevent bursting while allowing individual fast spikes [13]. We used the model simulations to investigate the inward currents during phases of the burst, including rapid depolarization, afterdepolarizing potential (ADP), and the wide spike prior to burst termination.

**Figure 4:**
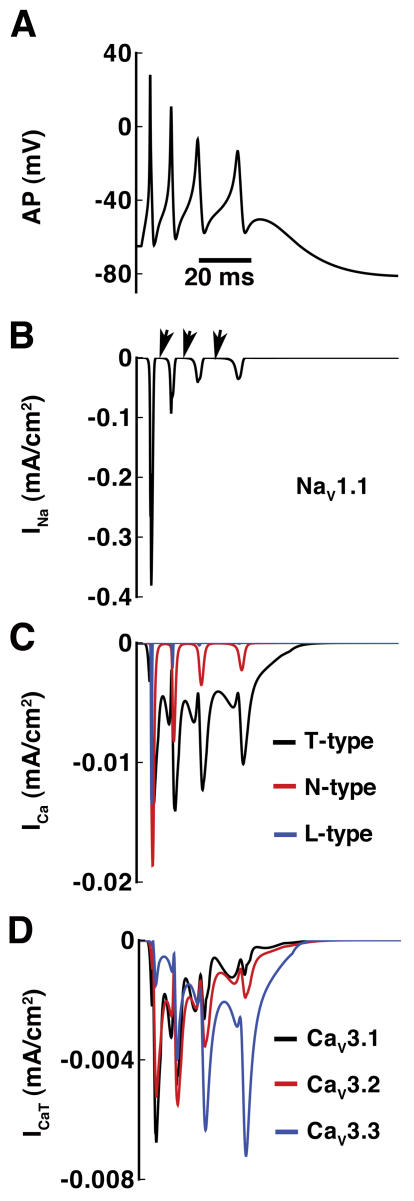
The ionic mechanism of bursting in the CA3 neuron. (A) The membrane potential in response to a brief current injection (0.2 nA for 5 ms). (B) The Na^+^ current in the neuron consists of Na_V_1.1 current. During the interspike interval, the current through Na_V_1.1 returns to zero (arrows). (C) T-type (black), N-type (red), and L-type (blue) Ca^2+^ currents during burst. Note that the L- and N-type Ca^2+^ currents are much smaller than the T-type Ca^2+^ current (black line) during the interspike intervals. (D) The three T-type Ca^2+^ current subtypes Ca_V_3.1, Ca_V_3.2 and Ca_V_3.3.

Figure 4A is a model generated somatic burst in response to a brief somatic stimulus (0.2 nA for 5 ms). Panels B-D are the primary depolarizing currents including the somatic Na^+^ current in (B) (Na_V_1.1), T-, N-, and L-type Ca^2+^ current (C) and the three T-type subtypes in (D). Currents are shown on the same time scale as the AP. Previous studies have suggested the importance of the afterdepolarizing potential (ADP) to sustain bursting in hippocampal neurons [13], [14]. Although Na_V_1.1 Na^+^ current contributes significantly to depolarization during the burst, it does not contribute to the afterdepolarizing potential (ADP) during the interspike interval, where the current fully deactivates (arrows in B). L- and N-type Ca^2+^ currents are much smaller than the T-type Ca^2+^ current (black line in C) during the interspike intervals. Indeed, T-type Ca^2+^ current is the primary contributor to the afterdepolarizing potential (ADP) and facilitates burst firing by boosting the afterdepolarizing potential (ADP) to firing threshold.

Subtype-specific T-type Ca^2+^ channel properties make distinct contributions to neuronal excitability and action potential morphology as shown in (D). Examination of individual T-subtypes in (D) shows that the two “faster” channels Ca_V_3.1 and Ca_V_3.2 contribute primarily early on in the burst via large inward currents that inactivate rapidly due to fast inactivation kinetics and close slowly due to their slow deactivation kinetics. Ca_V_3.3 contributes to sustained neuronal firing due to slow activation and inactivation kinetics. Ca_V_3.3 generates a small inward current initially, which gets larger with subsequent depolarization, so-called channel facilitation [29].

### The contribution of T- type Ca^2+^ channels to afterdepolarizing potential (ADP) and the burst generation in the soma

**Figure 5:**
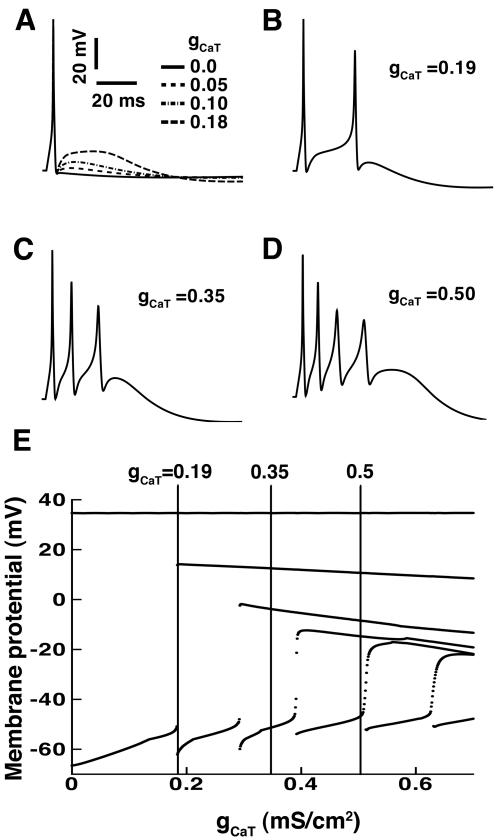
T-type Ca^2+^ channels underlie somatic afterdepolarizing potentials (ADP) and promote burst firing in the model neuron. Action potentials were elicited in response to a brief current of 0.2 nA for 5 ms. (A) Increasing g_CaT_ increases afterdepolarizing potential (ADP) in the model neuron. Shown are g_CaT_ (mS/cm^2^) = 0.0 (solid line), 0.05 (dashed line), 0.10 (dash-dot line), and 0.18 (long dash line), respectively. (B) When g_CaT = _0.19, a second spike is generated. Panels (C) and (D) correspond to the values of g_CaT_ = 0.35 and 0.5, respectively. (E) The effect of changing g_CaT_ on burst generation in the model neuron is shown for a range of g_CaT_ from 0.0 to 0.7 mS/cm^2^. Maximums in membrane potential (for each spike within a burst) generated at each value of g_CaT_ are shown. Lines are drawn vertically through values in panels B (g_CaT = _0.19, line intersects three points indicating three voltage maxima-two spikes and a wide spike prior to termination), C (g_CaT = _0.35, line intersects four voltage maxima-three spikes and a wide spike prior to termination) and D (g_CaT = _0.5, line intersects five points indicating five voltage maxima-four spikes and a wide spike prior to termination).

In order to probe the effects of T-type Ca^2+^ channels on afterdepolarizing potential (ADP) generation and bursting, we explored the effects of varying g_CaT_ as shown in Figure 5. For small g_CaT_ values (0∼0.18 mS/cm^2^), the cells failed to burst (Figure 5A). Rather, a single spike was followed by a larger afterdepolarizing potential (ADP) as g_CaT_ was increased. When g_CaT_ was increased to 0.19 mS/cm^2^, the afterdepolarizing potential (ADP) was large enough to bring Na^+^ channels to threshold and produce a second spike (Figure 5B). At g_CaT = _0.35 mS/cm^2^ and 0.5 mS/cm^2^ a burst is generated, and more spikes were observed with increasing g_CaT_ (Figure 5C and D). A summary of the effects of changing g_CaT_ on voltage dynamics in the model neuron is shown for a range of g_CaT_ from 0.0 to 0.7 mS/cm^2^ (Figure 5E). Voltage maximums indicating the number of spikes generated in a burst at each value of g_CaT_ are shown. Vertical lines are drawn through values corresponding to the voltage maxima recorded at values of g_CaT_ in panels B-D. At g_CaT = _0.19 (as in B), the vertical line intersects three points indicating three voltage maxima-two spikes and a wide spike prior to burst termination. At g_CaT = _0.35 (as in C) the line intersects four voltage maxima-three spikes and a wide spike prior to termination, while at g_CaT = _0.5, there is intersection with five points indicating five voltage maxima-four spikes and a wide spike prior to termination.

### Contribution of T- type Ca^2+^ channel subtypes

**Figure 6:**
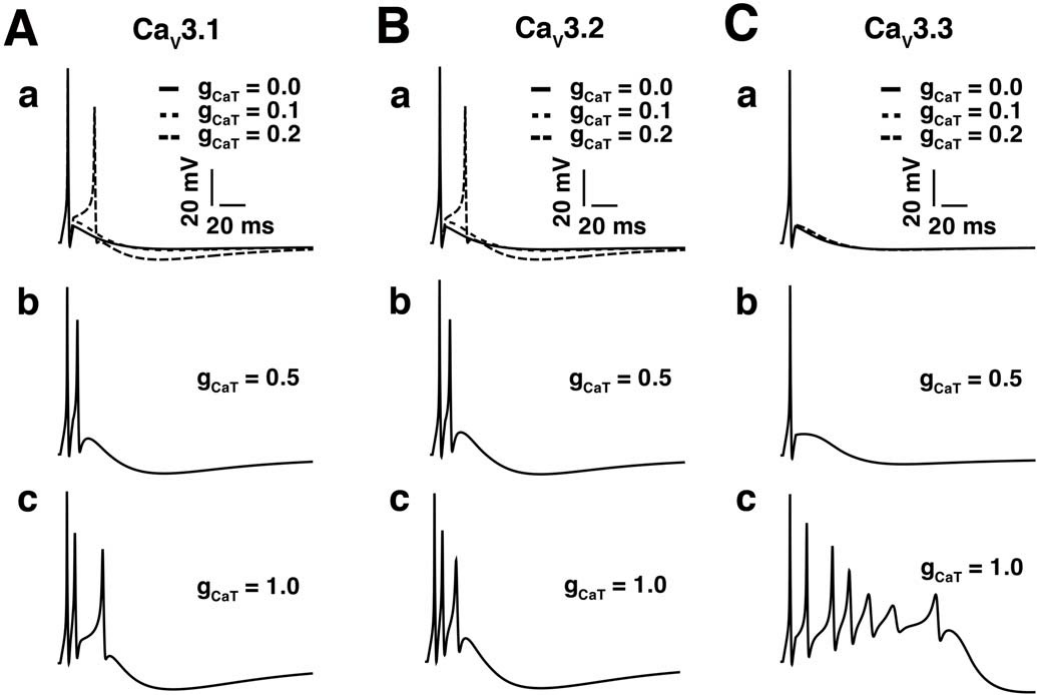
Effects of varying g_CaT_ for each T-type Ca^2+^ channel subtype. In each panel, the stimulus was 0.1 nA for 10 ms. Panels (a), (b), and (c) in columns A, B, and C show the effects of varying g_CaT_ for Ca_V_3.1, Ca_V_3.2, and Ca_V_3.3, respectively. (A) The effects of varying g_CaT_ for Ca_V_3.1: increasing g_CaT_ increases afterdepolarizing potential (ADP) and promotes bursting (in 6Aa, solid line for g_CaT_ = 0 versus short broken lines for 0.1 mS/cm^2^). With increasing g_CaT_, a burst emerges as shown in Figures 6Aa (long broken line) to 6Ac. (B) Similar results were obtained when Ca_V_3.2 channels were expressed alone. (C) The effects of varying g_CaT_ for Ca_V_3.3: the transition between a single spike (panel b for g_CaT = _0.5 mS/cm^2^) and a burst (panel c for g_CaT = _1.0 mS/cm^2^) is abrupt.

Our simulations suggest that T-type Ca^2+^ channels are primary contributors to the afterdepolarizing potential (ADP) that supports burst generation in our model neuron. We next explored more directly the role of each T-type Ca^2+^ channel subtypes in burst generation. We did so by testing whether each subtype alone had the potential to generate a burst, in order to determine if the unique kinetics of each channel are critical to produce the burst morphology, or if sufficiently large conductance of any one channel is enough to generate the burst. The effects of varying g_CaT_ of each subtype (expressed alone in the model neuron) are shown in Figure 6.

In Figure 6A, the effect of increasing g_CaT_ for Ca_V_3.1 is shown. As illustrated in Figure 6Aa, Ca_V_3.1 contributes to the afterdepolarizing potential (ADP) (solid line for g_CaT_ = 0 versus dotted line for 0.1 mS/cm^2^ and short broken lines for 0.2 mS/cm^2^). With increasing g_CaT_, a burst (albeit with an unusual morphology) emerges (Figure 6Aa–c). Similar results were obtained when Ca_V_3.2 channels were expressed alone in the model neuron except that spikes emerged slightly faster due to the slower inactivation kinetics of Ca_V_3.2 kinetics (Figure 6Ba–c).

In contrast, increasing Ca_V_3.3 g_CaT_ from 0.0 to 0.5 mS/cm^2^ causes an increase in the amplitude of the afterdepolarizing potential (ADP), but does not do so sufficiently to generate an additional spike (Figures 6Ca–b). However, when g_CaT_ was increased further to 1.0 mS/cm^2^, a typical burst was elicited (Figure 6Cc). The simulation suggests that the abrupt transition from single spike (Figure 6Cb) to complex burst firing (Figure 6Cc) is because there exists a threshold above which Ca_V_3.3 channels are self-facilitating. At relatively high g_CaT,_ large afterdepolarizing potentials (ADPs) are generated, which drives Ca_V_3.3 channel facilitation.

**Figure 7:**
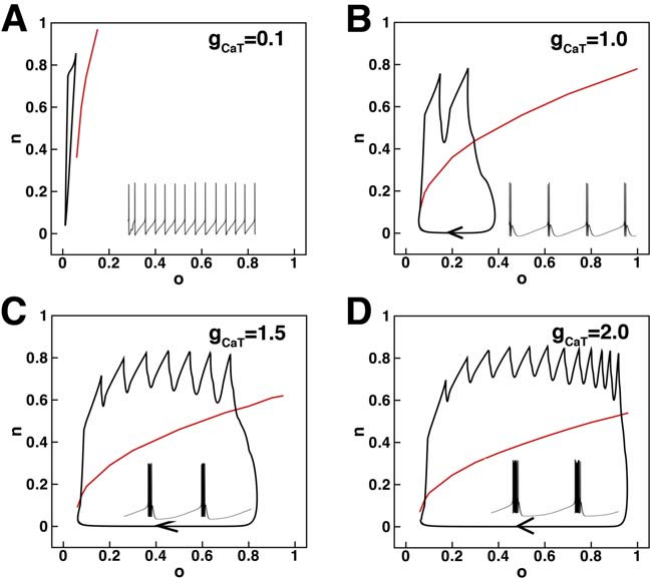
Fast slow analysis of bursting in a reduced CA3 model. The phase plot shows the projection of the burst trajectory onto the slow-variable n–o plane (n: the activation gate of Ca_V_3.1; o: the activation gate of Ca^2+^-dependent K^+^ channel). Direction of movement is indicated with arrows. Inset within each phase plot is the corresponding membrane potential timecourse over 500 ms. (A) g_CaT_ = 0.1 mS/cm^2^; (B) g_CaT_ = 1.0 mS/cm^2^; (C) g_CaT_ = 1.5 mS/cm^2^; (D) g_CaT_ = 2.0 mS/cm^2^. The red curve is the voltage nullcline (dV/dt = 0) of the fast subsystem with n and o as parameters. The region above the nullcline is the repetitive firing region. Below the curve is the silent region.

In order to develop a better quantitative understanding of the determinants of bursting, the interaction of ionic components in burst generation and the voltage dynamics with changes in isoform-specific g_CaT_, we performed fast-slow analysis in a reduced comparable model (details in Methods). In Figure 7 we show this analysis for Ca_V_3.1. The same analysis for Ca_V_3.2 and Ca_V_3.3 is contained in the online supplement (see Text S1, Figure S3, and Figure S4). The reduced model allows for separation of the variables into two subsystems: fast (fully responsive within the timescale of a spike) and slow (changes on the timescale of the whole burst). Unlike the Migliore model of CA3 and the recent model of CA1 from Golomb et al., which rely on window or persistent Na^+^ current to enhance bistability of the fast subsystem as a bursting mechanism (see [23]), our model burst is generated as a result of the interaction of two slow variables, a slow autocatalytic variable **n** (Ca_V_3.1 T-type Ca^2+^ channel activation gate) and a slow negative feedback variable **o** (Ca^2+^-dependent potassium (K^+^) channel activation gate); all other variables in our system are fast (and are insufficient to generate somatic bursts–see Figure 1C, middle panel). Figure 7, A–D: We first treated slow variables **n** and **o** as parameters to study the fast subsystem to determine regions of spiking and rest states for different values of g_CaT_ as indicated in (A) g_CaT_ = 0.1 mS/cm^2^; (B) g_CaT_ = 1.0 mS/cm^2^; (C) g_CaT_ = 1.5 mS/cm^2^; (D) g_CaT_ = 2.0 mS/cm^2^. The two-parameter phase plot shows the projection of the burst trajectory onto the slow variable **n**–**o** plane. The red line is the voltage nullcline (dV/dt = 0) for the fast system at the indicated values of **n** and **o** (where the saddle node bifurcation occurs–see [32] for details). The interaction of **n** and **o** generates slow oscillations that move the fast subsystem in and out of the repetitive spiking regime. The region above the nullcline corresponds to a region of repetitive firing and the region below the nullcline is where the system is silent. The direction of the trajectory is indicated with arrows.

### Effects of an epilepsy-linked mutation on cellular waveforms

**Figure 8:**
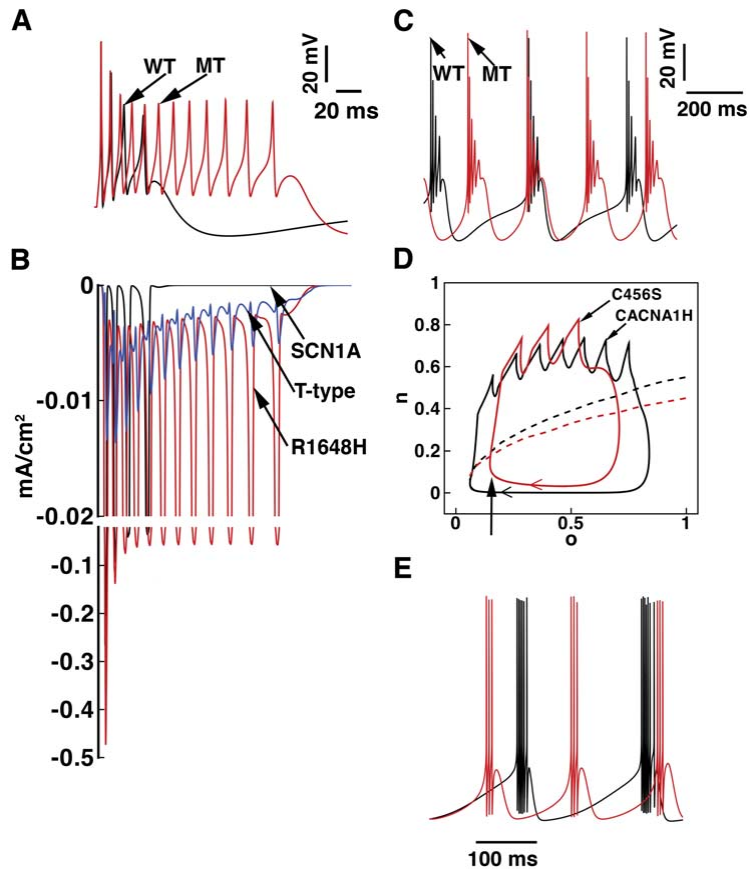
The effects of Na^+^ and T-type Ca^2+^ channel mutations on neuronal excitability. Panels A and B show the bursting behavior of the wild-type Na_V_1.1 and mutant R1648H model neurons. (A) Action potentials were generated in response to a brief (5 ms) 0.2 nA current injection for R1648H (red line) and wild-type Na_V_1.1 (black line). (B) The corresponding wild-type Na_V_1.1 and mutant R1648H Na^+^ current (on split scale). T-type Ca^2+^ current is also plotted for comparison. Note that the persistent Na^+^ current between spikes is much more prominent for R1648H compared with the wild-type. Panels C, D, and E show the bursting behavior of the wild-type Ca_V_3.2 and C456S single nucleotide polymorphism (SNP) model neurons. (C) Action potentials were generated in response to a steady 0.001 nA current injection for single nucleotide polymorphism (SNP) C456S (red line) and wild-type Ca_V_3.2 (black line) model neurons. The single nucleotide polymorphism (SNP) C456S increases the frequency of firing (e.g., reduces the intervals between bursts) (red line), compared to the wild type (black line). (D) Fast-slow analysis with wild-type Ca_V_3.2 (black) or single nucleotide polymorphism (SNP) C456S (red) channels in a reduced model. The phase plot shows the projection of the burst trajectory onto the slow variable n–o plane (n: the activation gate of Ca_V_3.2; o: the activation gate of Ca^2+^-dependent K^+^ channel). The dashed lines denote the stability line that separates resting states (below the line) from firing states (above the line). The arrows indicate the direction of the trajectory. (E) The action potentials corresponding to the parabolic burst firings as shown in B in the reduced model.

We next used the model to examine the effects of an epilepsy-linked Na_V_1.1 Na^+^ channel mutation, R1648H, on the cellular level action potential waveform. The R1648H mutation disrupts inactivation of the channel and results in a persistent non-inactivating current [6], [36] without significant changes in channel activation, availability, or recovery from inactivation [6]. We and others have previously developed models that recapitulate the gating properties of the mutation [7], [8], [20]. Figure 8 shows the effect of the mutation on the cellular electrical waveform. The wild-type neuron fired a typical burst (black line in A), while the R1648H mutant neuron produced a markedly prolonged burst (red line in A). Figure 8B shows the corresponding wild-type Na_V_1.1 and mutant R1648H currents. The R1648H mutant leads to an increase in Na^+^ current contributing both to the spiking phase of the burst and to the afterdepolarizing potential (ADP) in between spikes. In fact, the contribution of persistent Na^+^ current to the afterdepolarizing potential (ADP) is comparable with T-type Ca^2+^ current (blue line). Since Na^+^ influx does not directly result in an increase in ion-dependent K^+^ currents, spikes are generated until Ca^2+^ channels have sufficient time to inactivate and inward currents are overwhelmed by repolarizing currents. Thus, our results suggest that mutant R1648H channels can produce hyperexcitability, which manifests in single CA3 neurons as a prolonged endogenous burst duration.

We also used the model to probe the effect of C456S, one of the epilepsy-related single nucleotide polymorphisms (SNPs) in the gene CACNA1H that encodes the Ca_V_3.2 T-type Ca^2+^ channel subtype. This single nucleotide polymorphism (SNP) increases the rate of channel activation, observed as a −5 mV shift of the activation curve compared to the wild type. The SNP also results in a ∼1.6 fold increase in expression compared to wild type [10]. In our simulations, we replaced the wild type Ca_V_3.2 channel model with the C456S channel model, which incorporates the changes observed experimentally. The results are shown in Figsure 8C. In response to a continuous current injection of 0.001 nA, C456S current increased the frequency of burst firing (e.g., a reduction in the intervals between bursts) as shown in Figure 8C (red line), compared to the wild type (black line). Also, the firing threshold in the neuron with the C456S was lower compared with the wild type. These simulations suggest that C456S can also promote the hyperexcitability, but by an entirely different mechanism than the Na^+^ channel mutation, which promotes hyperexcitability by affecting the duration of the burst. The results of fast-slow analysis of the C456S single nucleotide polymorphism (SNP) are shown in Figure 8D. In the reduced model (as described for Figure 7) with the phase portrait of the projection of the burst trajectory onto the slow-variable **n**–**o** plane (**n**: the activation gate of Ca_V_3.2; **o**: the activation gate of Ca^2+^-dependent K^+^ channel), we observe a compression of the trajectory with the single nucleotide polymorphism (SNP). In a region of inhibition that is sufficient to suppress onset of a burst with wild-type channels, we observe transition in the mutant case into the repetitive firing region (arrow). This is a direct result of the mutation-induced shift of the activation gate of the Ca^2+^ channel (observed as a positive displacement of the phase portrait in the stable parameter regime). The net result of these changes is a shortening of the period of bursting observed in the time series in the reduced model (Figure 8E), consistent with the simulations in our full model (Figure 8C).

## Discussion

Bursting in CA3 neurons is critical for reliable transmission of electrical signals important for learning and memory, but also increases susceptibility to abnormal excitation that can cause seizures. In this study, we present a single-compartment computational framework where the goal is to reveal the contribution of important ionic components and their interaction to bursting in CA3 neurons.

### Mechanisms of CA3 bursting

We have incorporated descriptions of primary depolarizing currents in CA3 by constructing models for voltage-gated sodium (Na^+^) and calcium (Ca^2+^) currents that recapitulate recent experimentally measured channel kinetics. We then used our improved model to try to reveal the specific ionic mechanisms of bursting and temporal contribution of the primary depolarizing currents. We examined the contribution of inward currents during a burst, specifically contributions to the model-generated action potential spikes, afterdepolarizing potential (ADP), and the wide spike prior to burst termination. Our model neuron also successfully simulated rebound bursting, which previous models failed to reproduce. Indeed, Traub and Miles suggested that the failure of those models may be due to the insufficiently accurate representation of Na^+^ or low-voltage-activated Ca^2+^ currents [4].

### Na^+^ channels

Na^+^ channel subtypes play specialized roles in neuronal action potential excitability, firing frequency and morphology that are determined by their unique kinetics [37], [38]. Na_V_1.1, which has negligible persistent component, does not contribute directly to the afterdepolarizing potential (ADP). Rather, Na_V_1.1 causes rapid spike depolarization and then fully deactivates during the interspike interval (Figure 4).

### Ca^2+^ channels

Unlike regular spiking neurons, bursting neurons do not fully repolarize between Na^+^ mediated spikes. The repolarization that occurs between spikes becomes more incomplete with each spike, leading to a gradual inter-spike trend of increasing depolarization, the afterdepolarizing potential. In CA3 neurons, T-type Ca^2+^ channels have been suggested to play an important role in afterdepolarizing potential generation [11], [25], [26]. They have slow deactivation kinetics and close slowly during membrane repolarization, which may allow for a buildup of current during bursting [39]. The three T-channel subtypes present in CA3 neurons, Ca_V_3.1, Ca_V_3.2 and Ca_V_3.3, exhibit distinct functional properties [29], [39].

Both Ca_V_3.1 and Ca_V_3.2 contribute primarily early in the burst due to their similar kinetics. They activate at low voltages and then inactivate rapidly in response to depolarization. Slow deactivation kinetics ensures their contribution to the afterdepolarizing potential (ADP) between spikes, but a gradual buildup of inactivation results in “turning off” of the channels over the course of the burst. In contrast, Ca_V_3.3, with its slow activation and inactivation kinetics, is facilitated and contributes increasingly as the burst progresses. The current serves as an important partial contributor to spike broadening during the burst since it is the only depolarizing current that is facilitated in the subsequent spikes. Other studies have proposed that spike broadening results from cumulative inactivation of several potassium (K^+^) channels, including large-conductance (BK-type) Ca^2+^-dependent K^+^ channels [40], voltage-gated delayed rectifier K^+^ channels [41], and voltage-gated A-type K^+^ channels [42]. Presumably spike broadening may contribute to the enhanced transmitter release (and perhaps recruitment of post-synaptic neurons to network bursts) since spike broadening increases Ca^2+^ influx primarily by prolonging the open time of Ca^2+^ channels [42], [43]. Our simulations suggest that the burst is terminated when enough Ca^2+^ has entered through T-type channels to activate Ca^2+^-activated K^+^ channels, which overwhelm the Ca^2+^ current and terminate the burst.

### Fast-slow analysis of our system

In our CA3 model a burst is generated as a result of the interaction of two slow variables, a slow autocatalytic variable corresponding to the T-type Ca^2+^ channel activation gate and a slow negative feedback variable, the Ca^2+^-dependent K^+^ channel activation gate. This is in contrast to the Migliore model of CA3 [22], Traub model [18], [21] and the recent model of CA1 from Golomb et al., [23] in which bursting derives from bistability of the fast subsystem of the model. In those models, bistability is enhanced by large overlap in the activation and inactivation gates of the Na^+^ channel, which generates a large Na^+^ window current. It is, however, critical to question the physiological relevance of the fast bistable mechanism in these systems, since in all of these models channel availability is hugely shifted to depolarized potentials (>20 mV) compared to experimental measurements. It is exactly this shift that generates the window current and drives the instability.

In our system, the results of the fast slow analysis suggests that interaction of the T-type Ca^2+^ channel activation gate and the Ca^2+^-dependent K^+^ channel activation gate generates slow oscillations that move the fast subsystem from a stable non-oscillatory regime into a repetitive spiking regime and back again. Superimposition of the voltage nullcline (Figure 7) computed with the gates as parameters illustrates where this bifurcation occurs.

### Naturally occurring mutations affect cellular firing properties

Ion channel kinetics and conductance are determinants of neuronal cellular waveforms and perturbations in these properties affect neuronal electrical behavior. Mutations in genes encoding neuronal ion channels have been linked to genetic epilepsies and presumably cause deleterious outcomes in patients by causing defects in ion channel gating that disrupt cellular and network function [7]. Here we tested the effects of two of the mutations on cellular electrical behavior. The Na^+^ channel mutation, R1648H, has been modeled at the channel level previously by our group and others [7], [8], [20]. The primary consequence of R1648H is to disrupt inactivation, which results in a persistent non-inactivating current [6]. Our simulations demonstrated that R1648H results in a large additional inward current from non-inactivating Na^+^ channels contributing to the afterdepolarizing potential (ADP). The effect on the cell is a reduction in the interspike intervals and an increase in burst duration, characteristics of hyperexcitability. In the context of fast-slow analysis, an interesting plan for future studies is to explore the effect of R1648H on bistability of the fast subsystem in our model neuron. It will be important to determine the nature of the interaction between a bifurcation-generating bistable fast subsystem and the parabolic bursting mechanism that elicits bifurcations in our wild-type system.

We also investigated the effect of an epilepsy-associated Ca_V_3.2 single nucleotide polymorphism (SNP), C456S, on firing properties in the model neuron. The single nucleotide polymorphism hastens activation kinetics [9] and increases cell surface expression [10]. The effects of these changes are to increase excitability and bursting frequency in a single cell, properties that are preserved in the reduced model we used for fast-slow analysis.

### Conclusions

It is important to note that although we can use the model to simulate the effects of mutations on cellular level electrical activity, investigations at the cellular scale of the system do not provide information about the mechanism of the network burst associated with epilepsy. Because the epilepsy phenotype is fundamentally an emergent phenomenon that arises in networks, we cannot use the single cell model to glean meaningful mechanistic insights into network driven seizures. At this stage we can only show the effect of the mutation on the cellular level behavior and hypothesize that such deranged waveforms will increase the likelihood of synchronization under certain synaptically coupled conditions. Long term, we hope that our model may be used as a basic building block in the development of a virtual hippocampus that can be used to study the effects of natural and applied perturbations, such as mutations and drugs, on emergent dynamics of the hippocampus.

## Supporting Information

Text S1 Supplemental text
(0.11 MB DOC)

Table S1 Transition rates for Markov model NaV1.1 and R1648H
(0.05 MB DOC)

Table S2 Channel kinetics (Hodgkin-Huxley formalism)
(0.08 MB DOC)

Figure S1 The Markov model framework for Na+ channels. The model framework consists of 14 discrete states consisting of six closed states (UC3, UC2, UC1, LC3, LC2, and LC1), two conducting state (UO and LO), two closed-inactivation states (UIC3 and UIC2), two fast inactivation state (UIF and LIF), and two intermediate inactivation states (UIM1 and UIM2). The lower five states (prefixed with L) represent an additional mode of gating where channels fail to enter absorbing inactivation states and reopen. Channels in this mode produce persistent non-inactivating current that is prominent in the R1648H NaV1.1 mutant. More details can be found in [1].
(0.34 MB TIF)

Figure S2 Experimentally recorded (symbols) and simulated (lines) T-type Ca2+ channel kinetics. Computational models (solid lines) of CaV3.1 (red), CaV3.2 (blue), and CaV3.3 (black) T-type Ca2+ channel isofoms are shown and compared to experimental data from Chemin et al., 2002 (symbols). A: Activation curves were constructed by normalizing the peak current after depolarization from a holding potential of −110 mV to a range of potentials (−90 mV to −10 mV) to the largest peak current. Inactivation curves were obtained by normalizing peak currents obtained in response to a pulse to −30 mV after a long (500 ms until computed parameters are in steady-state) prepulse at indicated membrane potentials ranging from −110 mV to −30 mV. B: The time course of channel recovery from inactivation for each of the three isoforms is shown. Curves are for simulated and experimentally obtained currents using a two-pulse protocol. Peak currents elicited by the second pulse after the variable recovery period are normalized to peak current during first pulse. C: The time course of T-type Ca2+ currents for models and experiments are shown in response to a 100ms test pulse to −25 mV (black) or −35 mV (red and blue) from a holding potential of −110 mV. D: Model fits to experimentally recorded (symbols) normalized current during an action potential waveform clamp. Protocols are the same as in [10].
(0.23 MB TIF)

Figure S3 Fast slow analysis of bursting in a reduced CA3 model. The phase plot shows the projection of the burst trajectory onto the slow-variable n-o plane (n: the activation gate of CaV3.2; o: the activation gate of calcium-depend K+ channel). Direction of movement is indicated with arrows. (A) gCaT = 0.1 mS/cm2; (B) gCaT = 1.0 mS/cm2; (C) gCaT = 1.5 mS/cm2; (D) gCaT = 2.0 mS/cm2. The red curve is the voltage nullcline (dV/dt = 0) of the fast subsystem with n and o as parameters. The region above the nullcline is the unstable firing region. Below the curve is the stable rest region.
(0.53 MB TIF)

Figure S4 Fast slow analysis of bursting in a reduced CA3 model. The phase plot shows the projection of the burst trajectory onto the slow-variable n-o plane (n: the activation gate of CaV3.3; o: the activation gate of calcium-depend K+ channel). Direction of movement is indicated with arrows. (A) gCaT = 0.1 mS/cm2; (B) gCaT = 6.0 mS/cm2; (C) gCaT = 7.0 mS/cm2; (D) gCaT = 8.0 mS/cm2; (E) gCaT = 9.0 mS/cm2; (F) gCaT = 10.0 mS/cm2. The red curve is the voltage nullcline (dV/dt = 0) of the fast subsystem with n and o as parameters. The region above the nullcline is the unstable firing region. Below the curve is the stable rest region.
(0.69 MB TIF)
