# Supplementary material for: Ionic Mechanisms of Endogenous Bursting in CA3 Hippocampal Pyramidal Neurons: A Model Study
Source: PLoS One. 2008 Apr 30;3(4):e2056. doi: 10.1371/journal.pone.0002056 (PMC2323611; doi:10.1371/journal.pone.0002056)
Supplement: Text S1 — Supplemental text (0.11 MB DOC) [file pone.0002056.s001.doc]

**Supporting online material for "Ionic Mechanisms of Endogenous Bursting in CA3 Hippocampal Pyramidal Neurons: A Model Study"** by Jun Xu and Colleen E. Clancy

**Specific methods:**

**Na^+^ channels**

**Na^+^ channel model:** We have previously developed a model of the neuronal Na^+^ channel subtype Na_V_1.1, which we use here as shown in Figure S1 [1]. The Markov formulation of Na_V_1.1 I_Na_ is itself an extension of earlier modeling efforts by Horn and Vandenberg, Aldrich and Clancy and Rudy [2,3,4,5]. The Na_V_1.1 framework is a Markov model of fourteen coupled states that can reasonably recapitulate both dynamic and steady-state current properties [1]. This model is consistent with reported measurements of neuronal Na^+^ channel Na_V_1.1 encoded by gene SCN1A [6].

**T-type Ca^2+^ channels**

**T-type Ca^2+^ channel expression in CA3:** Previous studies have suggested that T-type channels are abundantly expressed in hippocampal CA3 pyramidal neurons [7,8] and a recent study showed that all three subtypes (Ca_V_3.1, Ca_V_3.2 and Ca_V_3.3) are expressed [9].

**T-type Ca^2+^ channel kinetics**: The T-type Ca^2+^ channel subtype kinetics is shown in Figure S2. Simulated currents are shown as lines and experimental data from Chemin et al. [10] as symbols. Figure S2A shows the activation constructed by normalizing the peak current after depolarization from a holding potential of -110 mV to a range of potentials (-90 mV to -10 mV) to the largest peak current, and inactivation obtained by stepping the membrane potential to -30 mV from a holding potential ranging from -110 to -30 mV. The simulated properties of the three subtypes agree well with experimentally observed behavior: Both steady-state activation and inactivation curves of Ca_V_3.3 subtype were shifted towards positive voltages, compared to the Ca_V_3.1 and Ca_V_3.2 subtypes. Figure S2B is the time course of recovery from inactivation obtained using two -30 mV test potentials. Peak currents elicited by the second pulse after the variable recovery period is normalized to the peak current during first pulse. Although the difference between the activation and inactivation kinetics for subtypes Ca_V_3.1 and Ca_V_3.2 are not significant as shown in Figure S2A, the recovery from inactivation for Ca_V_3.1 is noticeably distinct from that of Ca_V_3.2.

Figure S2C illustrates the macroscopic currents elicited by a 100 ms test pulse to -25 mV (Ca_V_3.3) or -35 mV (Ca_V_3.1 and Ca_V_3.2) from a holding potential of -110 mV. In the Ca_V_3.1 and Ca_V_3.2 channels, depolarization to -35 mV from a holding potential of -110 mV results in large macroscopic currents that fully inactivate and nearly decay to the zero current level. In contrast, Ca_V_3.3 current is marked by slower activation onset and decay as shown in Figure S2C. Finally, an action potential voltage clamp waveform was imposed to evaluate the time course of the three subtypes under conditions of changing voltage as might be observed during the action potential. Figure S2D showed the normalized current for the three subunits during an action potential clamp. From steady state at a holding potential of -76 mV, simulated cells were gradually depolarized to 35 mV followed by gradual repolarization to -76 mV. Three subtypes displayed distinct behavior during a single AP waveform. Ca_V_3.1 and Ca_V_3.2 produced large and slowly inactivating currents. In contrast, Ca_V_3.3 produced a faster inactivating current.

**FAST-SLOW ANALYSIS**

Fast-slow analysis was used to investigate the bursting mechanism in a reduced single-compartment (soma) CA3 model. See details in Methods. The fast-slow analysis for Ca_V_3.2 and Ca_V_3.3 is shown in Figure S3 and Figure S4, respectively.

**MOdel Parameters**

**Na^+^ channels**

Macroscopic current density is computed by

Where G_Na_ is the maximum membrane conductance density, P_O_ is the sum of all channel open probabilities, V is the membrane potential, and E_Na_ is the reversal potential. Transition rates are shown in Table S1.

**T-type Ca^2+^ channels**

To characterize the T-type Ca^2+^ channels, we re-fit kinetic models of three subtypes [10]. Using the Hodgkin-Huxley formalism, the change of gating variables with time (e.g., x) can be determined by solving the differential equation

where x_∞_ is the steady-state value and τ_x_ is the time constant.

The steady-state activation and inactivation were modeled with Boltzmann equation:

where V is the membrane potential, V_1/2_ is the voltage of half (in)activation, and k is the slope factor. T-type Ca^2+^ current was calculated using Goldman-Hodgkin-Katz (GHK) constant-field equation:

where $\tilde{g}_{\text{Ca}}$ is the maximum conductance, P_Ca_ is the permeability, z is the valence of Ca^2+^ (z=2), F is Faraday constant, R is the gas constant, T is the temperature, [Ca^2+^]_i_ and [Ca^2+^]_o_ are the intracellular and extracellular concentrations of Ca^2+^, respectively. Model parameters for T-type Ca^2+^ channels as well as all other channels in Hodgkin-Huxley formalism are shown in Table S2.

**References**

1. Clancy CE, Kass RS (2004) Theoretical investigation of the neuronal Na+ channel SCN1A: abnormal gating and epilepsy. Biophys J 86: 2606-2614.

2. Vandenberg CA, Horn R (1984) Inactivation viewed through single sodium channels. J Gen Physiol 84: 535-564.

3. Clancy CE, Rudy Y (1999) Linking a genetic defect to its cellular phenotype in a cardiac arrhythmia. Nature 400: 566-569.

4. Aldrich RW, Corey DP, Stevens CF (1983) A reinterpretation of mammalian sodium channel gating based on single channel recording. Nature 306: 436-441.

5. Horn R, Vandenberg CA (1984) Statistical properties of single sodium channels. J Gen Physiol 84: 505-534.

6. Lossin C, Wang DW, Rhodes TH, Vanoye CG, George AL, Jr. (2002) Molecular basis of an inherited epilepsy. Neuron 34: 877-884.

7. Fisher RE, Gray R, Johnston D (1990) Properties and distribution of single voltage-gated calcium channels in adult hippocampal neurons. J Neurophysiol 64: 91-104.

8. Kavalali ET, Zhuo M, Bito H, Tsien RW (1997) Dendritic Ca2+ channels characterized by recordings from isolated hippocampal dendritic segments. Neuron 18: 651-663.

9. Talley EM, Cribbs LL, Lee JH, Daud A, Perez-Reyes E, et al. (1999) Differential distribution of three members of a gene family encoding low voltage-activated (T-type) calcium channels. J Neurosci 19: 1895-1911.

10. Chemin J, Monteil A, Perez-Reyes E, Bourinet E, Nargeot J, et al. (2002) Specific contribution of human T-type calcium channel isotypes (alpha(1G), alpha(1H) and alpha(1I)) to neuronal excitability. J Physiol 540: 3-14.

11. Jaffe DB, Ross WN, Lisman JE, Lasser-Ross N, Miyakawa H, et al. (1994) A model for dendritic Ca2+ accumulation in hippocampal pyramidal neurons based on fluorescence imaging measurements. J Neurophysiol 71: 1065-1077.

12. Gold C, Henze DA, Koch C, Buzsaki G (2006) On the origin of the extracellular action potential waveform: A modeling study. J Neurophysiol 95: 3113-3128.

13. Migliore M, Cook EP, Jaffe DB, Turner DA, Johnston D (1995) Computer simulations of morphologically reconstructed CA3 hippocampal neurons. J Neurophysiol 73: 1157-1168.
