# Supplementary material for: Ionic Mechanisms of Endogenous Bursting in CA3 Hippocampal Pyramidal Neurons: A Model Study
Source: PLoS One. 2008 Apr 30;3(4):e2056. doi: 10.1371/journal.pone.0002056 (PMC2323611; doi:10.1371/journal.pone.0002056)
Supplement: Table S1 — Transition rates for Markov model NaV1.1 and R1648H (0.05 MB DOC) [file pone.0002056.s002.doc]

| **Rates** | **Na_V_1.1** | **R1648H** |
| --- | --- | --- |
| **α11** | 2.802/(0.21×exp(-v/17.0) +0.23×exp(-v/150)) | 2.802/(0.21×exp(-v/17.0) +0.23×exp(-v/150)) |
| **α12** | 2.802/(0.23×exp(-v/15.0) +0.25×exp(-v/150)) | 2.802/(0.23×exp(-v/15.0) +0.25×exp(-v/150)) |
| **α13** | 2.802/(0.25×exp(-v/12.0) +0.27×exp(-v/150)) | 2.802/(0.25×exp(-v/12.0) +0.27×exp(-v/150)) |
| **β11** | 0.4×exp(-v/20.3) | 0.4×exp(-v/20.3) |
| **β12** | 0.4×exp(-(v-5)/20.3) | 0.4×exp(-(v-5)/20.3) |
| **β13** | 0.4×exp(-(v-10)/20.3)/4.5 | 0.4×exp(-(v-10)/20.3)/4.5 |
| **α2** | 9.178×exp(v/29.68)/4.5 | 9.178×exp(v/29.68)/4.5 |
| **β2** | (α13×α2×α3)/(β13×β3) | (α13×α2×α3)/(β13×β3) |
| **α3** | (3.7933e-7×exp(-v/7.6))×3 | (3.7933e-7×exp(-v/7.6))×3 |
| **β3** | 0.0084+.00002×v | 0.0084+.00002×v |
| **α4** | (α2/100)×1.5 | (α2/100)×1.5 |
| **β4** | α3/5 | α3/5 |
| **α5** | (α2/95000)×80.0 | (α2/95000)×80.0 |
| **β5** | (α3/30)/10.0 | (α3/30)/10.0 |
| **αα2** | 0 | (9.178×exp(v/29.68))/4.5 |
| **ββ2** | 0 | (α13×αα 2×αα3)/(β13×ββ3) |
| **αα3** | 0 | (3.7933e-7×exp(-v/7.7))×83 |
| **ββ3** | 0 | 0.0084+.00002×v |
| **μ1** | 0 | 2e-5 |
| **μ2** | 0 | 2e-4 |
| **References** | [1] | [1] |
