# Supplementary material for: Ionic Mechanisms of Endogenous Bursting in CA3 Hippocampal Pyramidal Neurons: A Model Study
Source: PLoS One. 2008 Apr 30;3(4):e2056. doi: 10.1371/journal.pone.0002056 (PMC2323611; doi:10.1371/journal.pone.0002056)
Supplement: Table S2 — Channel kinetics (Hodgkin-Huxley formalism) (0.08 MB DOC) [file pone.0002056.s003.doc]

| **Ion channel** | **Kinetics** | **References** |
| --- | --- | --- |
| **T-type Ca^2+^ channels** | Ca_V_3.1  n_∞_=1/(1+exp(-(v+49.3)/4.6))  τ_n_= 0.8+0.025exp(-v/14.5) if v≥-56 mV  τ_n_= 1.71exp((v+120)/38) if v<-56 mV  l_∞_=1/(1+exp((v+74.2)/5.5))  τ_l_= 12.3+0.12exp(-v/10.8) if v≥-60 mV  τ_l_= 137 if v<-60 mV | Expt data from [10] |
|  | Ca_V_3.2  n_∞_=1/(1+exp(-(v+48.4)/5.2))  τ_n_= 1.34+0.035exp(-v/11.8) if v≥-56 mV  τ_n_= 2.44exp((v+120)/40) if v<-56 mV  l_∞_=1/(1+exp((v+75.6)/6.2))  τ_l_= 18.3+0.005exp(-v/6.2) if v≥-60 mV  τ_l_= 500 if v<-60 mV |  |
|  | Ca_V_3.3  n_∞_=1/(1+exp(-(v+41.5)/6.2))  τ_n_= 7.2+0.02exp(-v/14.7) if v≥-60 mV  τ_n_= 0.875exp((v+120)/41) if v<-60 mV  l_∞_=1/(1+exp((v+69.8)/6.1))  τ_l_= 79.5+2.0exp(-v/9.3) if v≥-60 mV  τ_l_= 260 if v<-60 mV |  |
| **N-type Ca^2+^ channel** | I_CaN_=g_CaN_×m^2^hv(1-[Ca^2+^]_i_/[Ca^2+^]_o_exp(2Fv/kT))/(1-exp(2Fv/kT))  α_m_ = 0.1967(-v+19.88)/(exp((-v+19.88)/10)-1)  β_m_ = 0.046exp(-v/20.73)  α_h_ = 1.6e-4exp(-v/48.4)  β_h_ = 1/(exp((-v+39)/10)+1) | [11] |
| **L-type Ca^2+^ channel** | I_CaL_=g_CaL_×m^2^v(1-[Ca^2+^]_i_/[Ca^2+^]_o_exp(2Fv/kT))/(1-exp(2Fv/kT))  α_m_ = 15.69(-v+81.5)/(exp((-v+81.5)/10)-1)  β_m_ = 0.29exp(-v/10.86) | [11] |
| **Ca^2+^ dependent AHP K^+^ channel** | I_AHP_=g_AHP_×w^8^(v-v_k_)  τ_w_ = 1/(1e31[Ca^2+^]^2^+0.2)+5.0  w_∞_= 1e31[Ca^2+^]2/(1e31[Ca^2+^]^2^+0.2) | [12] |
| **Ca^2+^ dependent K^+^ channel** | I_KC_ =g_KC_×o(v-v_k_)  α_o_ = [Ca^2+^]×0.28/([Ca^2+^]+0.48e-3exp( -2×0.84v×F/(RT)));  β_o_ = 0.48/(1+[Ca^2+^]/(0.13e-6exp(-2v×F/(RT)))) | [13] |
| **Delayed rectifier K^+^ channel** | I_KDR_=g_KDR_×n^3^×l×(v-v_k_)  α_n_=0.03exp(5×0.4(v+32)F/RT)  β_n_=0.03exp(-5×0.6(v+32)F/RT)  α_l_=0.001exp(-2(v+61)F/RT)  β_l_=0.001 | [13] |
| **A-type K^+^ channel** | I_KA_=g_KA_×nl(v-v_k_)  α_n_=0.02exp(3×0.6(v+33.6)F/RT)  β_n_=0.02exp(-1.2(v+33.6)F/RT)  α_l_=0.08exp(4(v+83)F/RT)  β_l_=0.08 | [13] |
| **Muscarinic K^+^ channel** | I_KM_=g_KM_×m(v-v_k_)  α_m_=0.006exp(10×0.06(v+55)F/RT)  β_m_=0.006exp(-9.4(v+55)F/RT) | [13] |
