# Supplementary figures and images for: Ionic Mechanisms of Endogenous Bursting in CA3 Hippocampal Pyramidal Neurons: A Model Study
Source: PLoS One. 2008 Apr 30;3(4):e2056. doi: 10.1371/journal.pone.0002056 (PMC2323611; doi:10.1371/journal.pone.0002056)

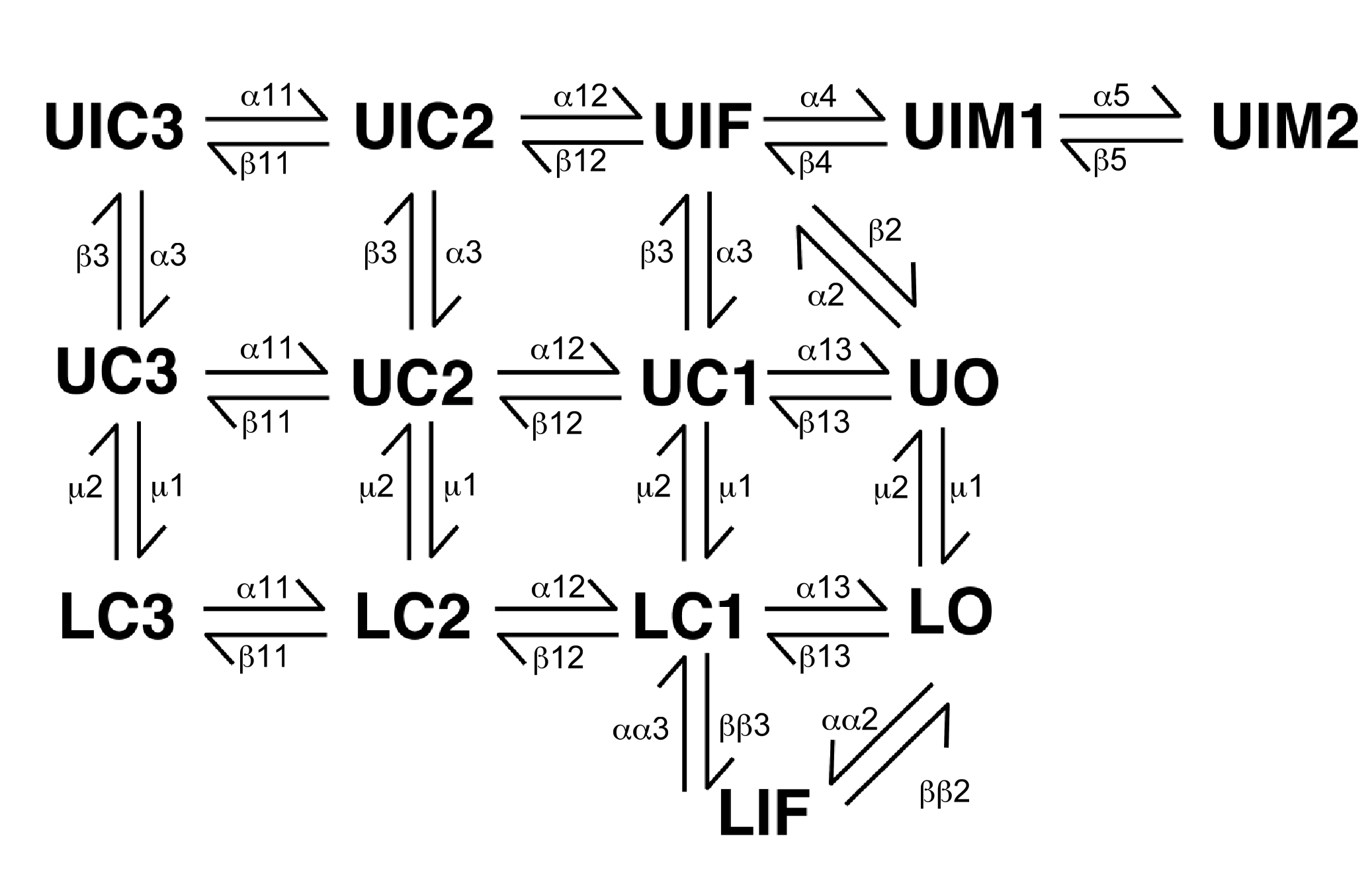

Supplement: Figure S1 — The Markov model framework for Na+ channels. The model framework consists of 14 discrete states consisting of six closed states (UC3, UC2, UC1, LC3, LC2, and LC1), two conducting state (UO and LO), two closed-inactivation states (UIC3 and UIC2), two fast inactivation state (UIF and LIF), and two intermediate inactivation states (UIM1 and UIM2). The lower five states (prefixed with L) represent an additional mode of gating where channels fail to enter absorbing inactivation states and reopen. Channels in this mode produce persistent non-inactivating current that is prominent in the R1648H NaV1.1 mutant. More details can be found in [1]. (0.34 MB TIF) [file pone.0002056.s004.tif]

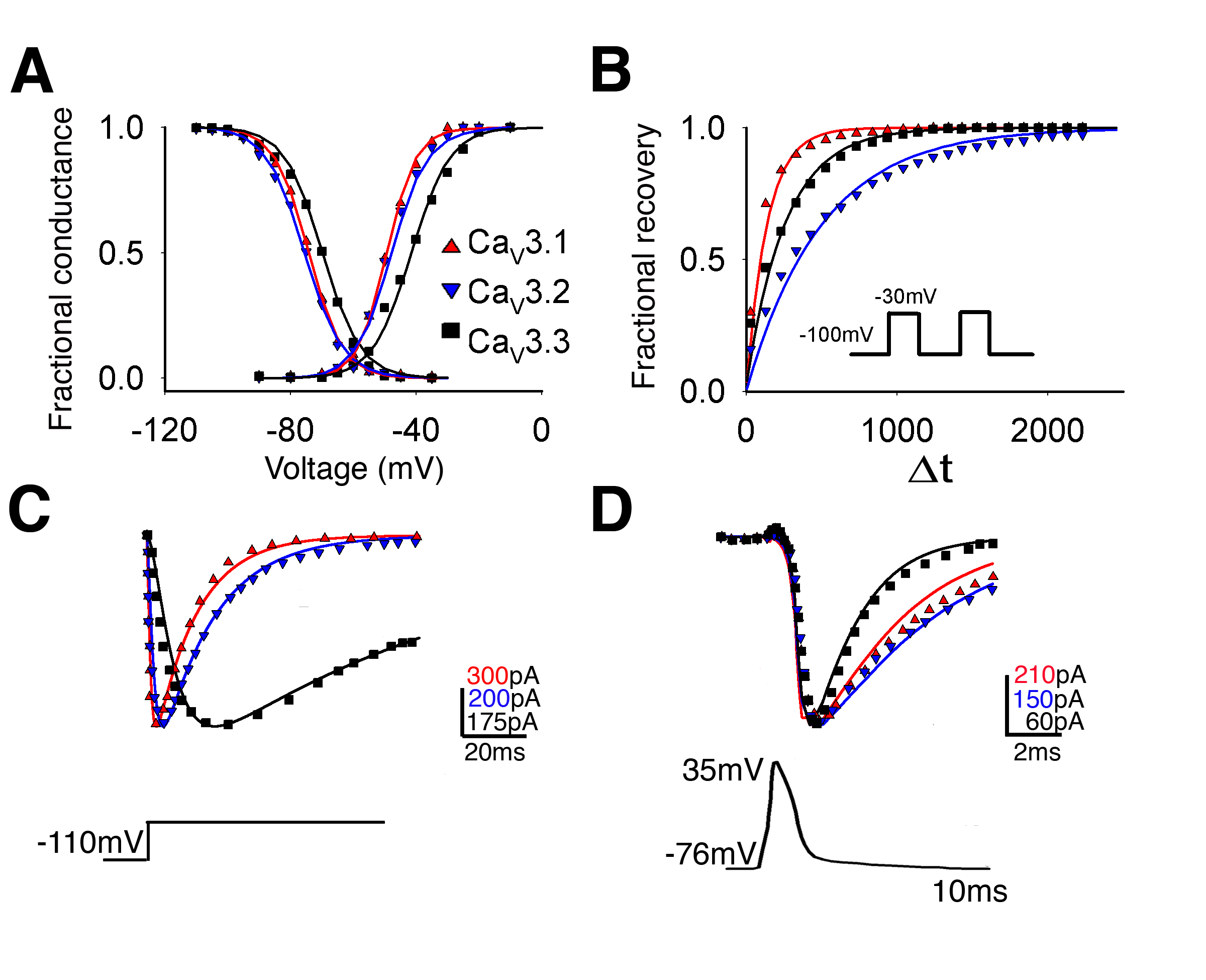

Supplement: Figure S2 — Experimentally recorded (symbols) and simulated (lines) T-type Ca2+ channel kinetics. Computational models (solid lines) of CaV3.1 (red), CaV3.2 (blue), and CaV3.3 (black) T-type Ca2+ channel isofoms are shown and compared to experimental data from Chemin et al., 2002 (symbols). A: Activation curves were constructed by normalizing the peak current after depolarization from a holding potential of −110 mV to a range of potentials (−90 mV to −10 mV) to the largest peak current. Inactivation curves were obtained by normalizing peak currents obtained in response to a pulse to −30 mV after a long (500 ms until computed parameters are in steady-state) prepulse at indicated membrane potentials ranging from −110 mV to −30 mV. B: The time course of channel recovery from inactivation for each of the three isoforms is shown. Curves are for simulated and experimentally obtained currents using a two-pulse protocol. Peak currents elicited by the second pulse after the variable recovery period are normalized to peak current during first pulse. C: The time course of T-type Ca2+ currents for models and experiments are shown in response to a 100ms test pulse to −25 mV (black) or −35 mV (red and blue) from a holding potential of −110 mV. D: Model fits to experimentally recorded (symbols) normalized current during an action potential waveform clamp. Protocols are the same as in [10]. (0.23 MB TIF) [file pone.0002056.s005.tif]

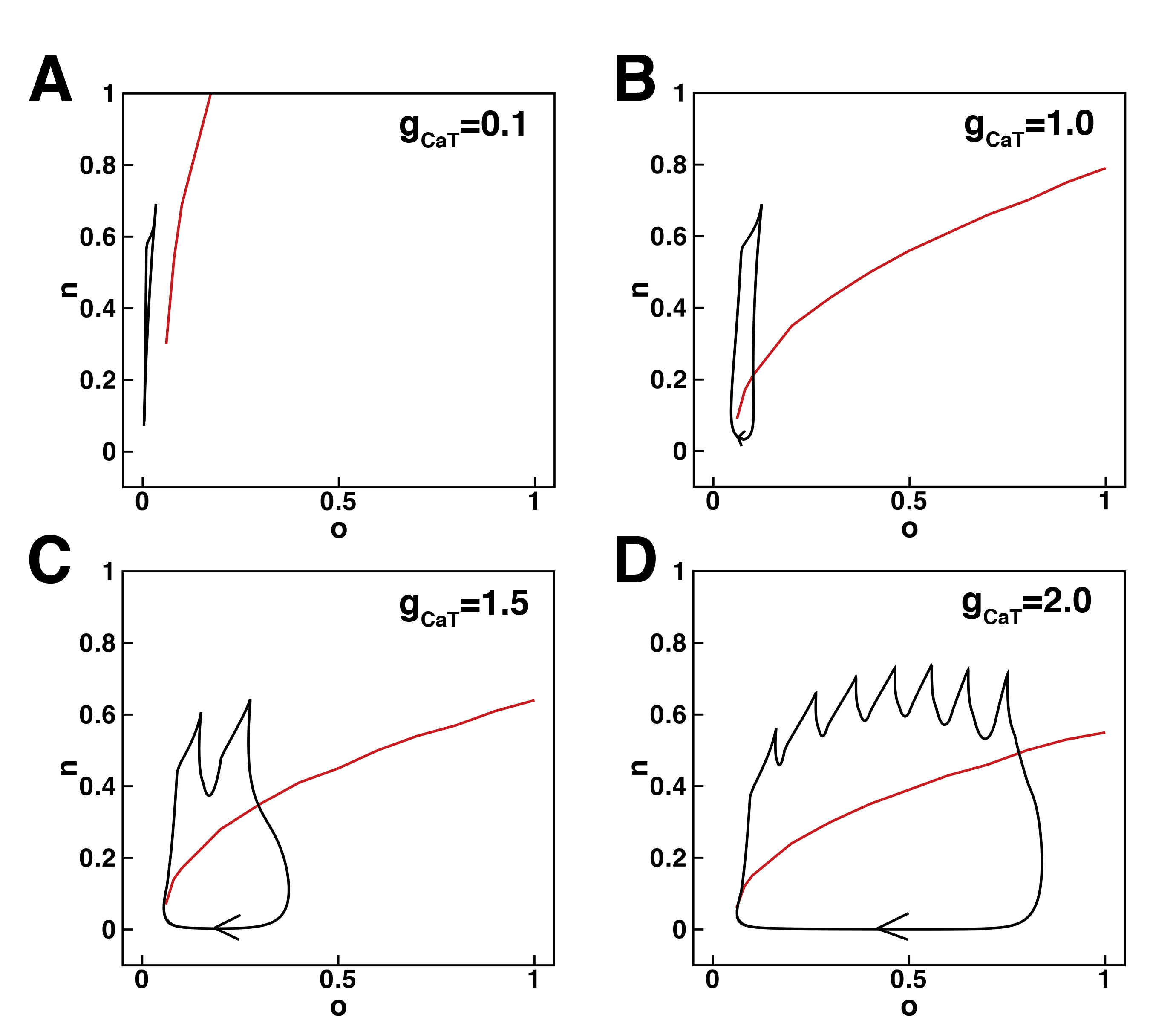

Supplement: Figure S3 — Fast slow analysis of bursting in a reduced CA3 model. The phase plot shows the projection of the burst trajectory onto the slow-variable n-o plane (n: the activation gate of CaV3.2; o: the activation gate of calcium-depend K+ channel). Direction of movement is indicated with arrows. (A) gCaT = 0.1 mS/cm2; (B) gCaT = 1.0 mS/cm2; (C) gCaT = 1.5 mS/cm2; (D) gCaT = 2.0 mS/cm2. The red curve is the voltage nullcline (dV/dt = 0) of the fast subsystem with n and o as parameters. The region above the nullcline is the unstable firing region. Below the curve is the stable rest region. (0.53 MB TIF) [file pone.0002056.s006.tif]

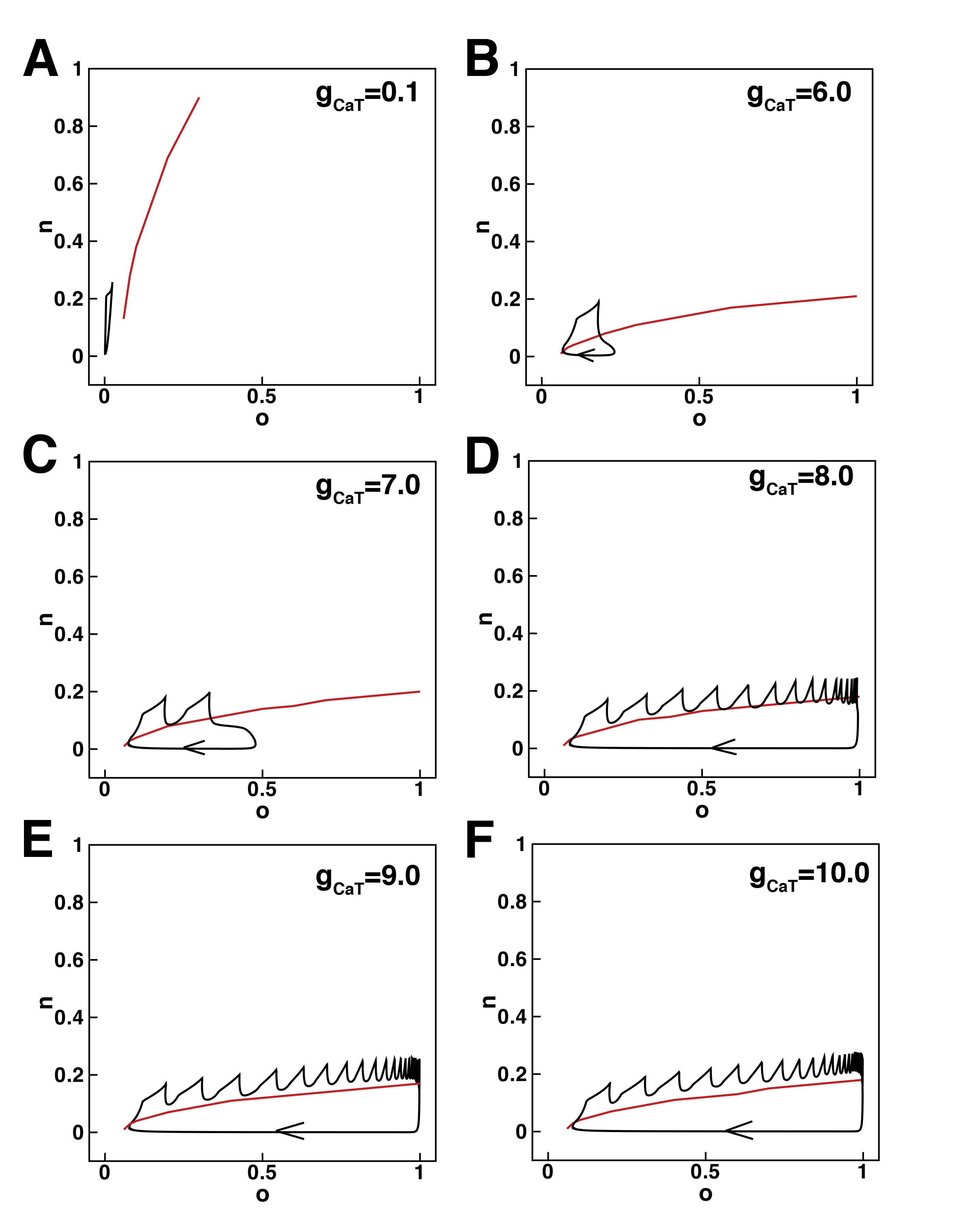

Supplement: Figure S4 — Fast slow analysis of bursting in a reduced CA3 model. The phase plot shows the projection of the burst trajectory onto the slow-variable n-o plane (n: the activation gate of CaV3.3; o: the activation gate of calcium-depend K+ channel). Direction of movement is indicated with arrows. (A) gCaT = 0.1 mS/cm2; (B) gCaT = 6.0 mS/cm2; (C) gCaT = 7.0 mS/cm2; (D) gCaT = 8.0 mS/cm2; (E) gCaT = 9.0 mS/cm2; (F) gCaT = 10.0 mS/cm2. The red curve is the voltage nullcline (dV/dt = 0) of the fast subsystem with n and o as parameters. The region above the nullcline is the unstable firing region. Below the curve is the stable rest region. (0.69 MB TIF) [file pone.0002056.s007.tif]
